# Something New

**Published:** 1863-07

**Authors:** 


### SOMETHING NEW.

We have just received from Dr. Bidwell, of Michigan several sample pieces—full upper dentures, the peculiarity of which, is in the arrangement of the chamber. The chamber is covered with non-vulcanizable rubber, which is claimed to be a great improvement. We have had no experience with it yet, but will test it as soon as convenient. It will, doubtless, answer well in some cases, and perhaps for all, where chambers are required. See his statement in the advertising sheet. We may have something farther to say after trying it. T.

